# Progress in Research on Metal Ion Crosslinking Alginate-Based Gels

**DOI:** 10.3390/gels11010016

**Published:** 2024-12-27

**Authors:** Yantao Wang, Zhenpeng Shen, Huili Wang, Zhaoping Song, Dehai Yu, Guodong Li, Xiaona Liu, Wenxia Liu

**Affiliations:** State Key Laboratory of Biobased Materials and Green Papermaking, Qilu University of Technology, Jinan 250353, China; wang272214@163.com (Y.W.); szp1542195405@163.com (Z.S.); zsong@qlu.edu.cn (Z.S.); yudehai@qlu.edu.cn (D.Y.); lgd@qlu.edu.cn (G.L.); lxn511944@qlu.edu.cn (X.L.); liuwenxia@qlu.edu.cn (W.L.)

**Keywords:** metal ion, alginate, gel, application

## Abstract

Alginate is an important natural biopolymer and metal ion-induced gelation is one of its most significant functional properties. Alginate-based hydrogels crosslinked with metal ions are commonly utilized in the food, biomedical, tissue engineering, and environment fields. The process of metal ion-induced alginate gelation has been the subject of thorough research over the last few decades. This review aims to summarize the mechanisms of alginate hydrogels induced by different cations (primarily including Ca^2+^, Ba^2+^, Cu^2+^, Sr^2+^, Fe^2+^/Fe^3+^, and Al^3+^). Metal ion-induced alginate gelation shows different preferences for α-L-guluronic acid (G), β-D-mannuronic acid (M), and GM blocks. Some metal ions can also selectively bind to the carboxyl groups of guluronic acid. The properties and applications of these alginate-based hydrogels are also discussed. The primary objective of this review is to provide useful information for exploring the practical applications of alginate.

## 1. Introduction

**Figure 1:**
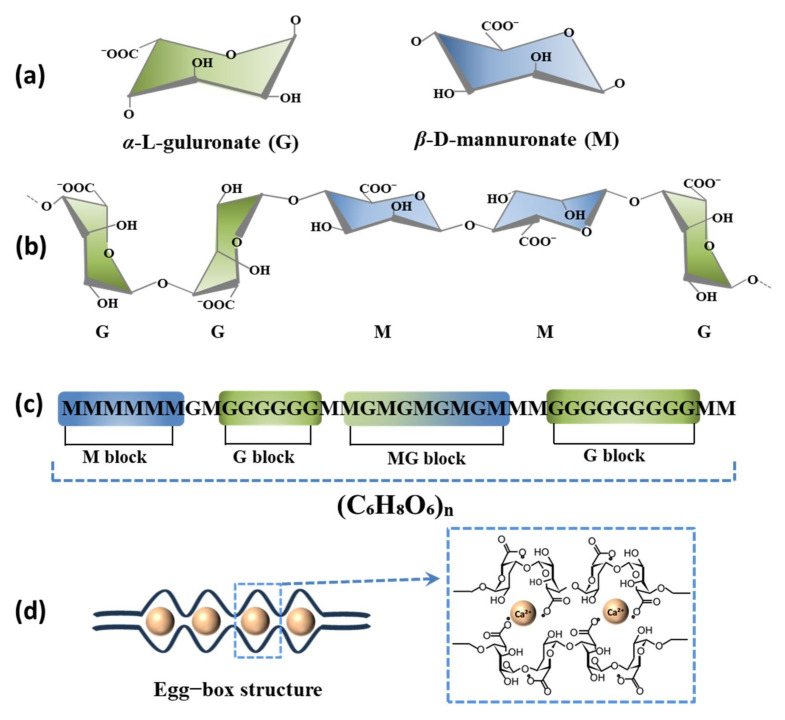
Structural characteristics of alginates. (**a**) Monomer units; (**b**) Chain conformation; (**c**) Block distribution; (**d**) Egg-box structure.

Alginates, anionic polysaccharides predominantly found in brown seaweed cell walls, have garnered attention in tissue engineering, wound dressings, and drug delivery materials for their non-toxicity, biocompatibility, and biodegradability [1,2]. Structurally, alginates are linear copolymers comprising (1–4)-linked β-D-mannuronic acid (M) and α-L-guluronic acid (G) residues [3,4]. These monomers are arranged sequentially in repeating or alternating blocks with varying proportions and sequences. Blocks of M, G, and mixed M/G-domains are distributed randomly along alginate polymer chains, as illustrated in Figure 1 [5]. Both types of building blocks contain carboxylic acid residues capable of binding to positively charged counterions. The effectiveness of ionic crosslinking in forming stronger connections is attributed to the differing conformations of M and G blocks. The robustness of alginate gel networks and their mechanical properties rely on the G/M ratio and the length of the G blocks [6]. Alginate from different bacterial sources is characterized by unique G/M ratios, quantifiable via NMR techniques.

Numerous studies have demonstrated that the carboxyl groups of alginate residues interact swiftly with metal ions, such as Ca^2+^, leading to the formation of a three-dimensional gel network structure, known as the “egg-box” structure [7]. The ion-induced gelation of alginate is a crucial functional property, underpinning numerous advantageous applications of this biomaterial. The preparation method is simple and user-friendly, and alginate gels possess many exceptional properties, such as biodegradability, tunable mechanical properties, and mild gelation conditions. These features offer great potential for widespread applications in biomedicine, environmental management, tissue engineering, and beyond [8]. However, the formation of alginate gels with these cations exhibits both similarities and differences in their gel mechanism, strength, and biocompatibility. Alginate-based hydrogels can have their network structure and mechanical properties regulated by metal ions. Therefore, it is necessary to discuss and compare the variances in the gelation mechanisms of alginate induced by different metal ions.

## 2. Preparation of Alginate-Based Hydrogels

### 2.1. Preparation Methods

The most common method for preparing alginate hydrogels is ionic crosslinking, particularly using one or two metal cations like Ca^2+^, Ba^2+^, and Al^3+^. The process is simple and presents the advantage of being quick, reversible, and devoid of the necessity for supplementary initiators and crosslinkers that may carry physiological toxicity, making it suitable for cell encapsulation and tissue engineering applications [9,10]. Ionic gelation methods mainly consist of external gelation and internal gelation. External gelation involves dripping alginate solution into a solution containing metal ions to form a gel through ion exchange [11]. Due to rapid gelation, the gel particle exhibits an inhomogeneous structure with an ion concentration gradient in thickness, leading to incomplete crosslinking in the inner core [12]. Internal gelation, on the other hand, entails adding insoluble metal salts and a buffering agent to the alginate solution and achieving gelation by adjusting the pH [12,13]. The internal method avoids the problem of ion concentration gradients and slow release and diffusion of metal ions, leading to the formation of a uniform gel structure [13].

Chemical crosslinking provides stronger and more stable hydrogels compared to ionic crosslinking. Common chemical crosslinkers include N,N′-methylenebisacrylamide (MBA) and glutaraldehyde (GA) [14]. The mechanical properties and degradation rates can be controlled by adjusting the crosslinker concentration and reaction conditions [15]. However, the preparation of alginate gels using chemical crosslinking methods is plagued by issues such as complexity, environmental pollution, and irreversibility. Certain crosslinking agents, like GA, may possess toxicity, thereby limiting their biomedical applications. The preparation process is intricate, requiring strict control of reaction conditions. Undesirable byproducts may be generated during the crosslinking process, impacting the environment. Chemical crosslinking is typically irreversible, posing challenges for post-use adjustments or recycling efforts.

Alginate, along with other polymers, can be used to create hybrid hydrogels that exhibit high mechanical strength, functionality, and excellent resistance to swelling. Functional polymers, such as gelatin and polyvinyl alcohol, have been used to prepare hybrid hydrogels with specific biological activity, antimicrobial properties, or drug release characteristics to gels [16]. Kim et al. developed gelatin-alginate hybrid hydrogels that exhibit strong resistance to external stimuli, including shear force, swelling, and temperature, for the purpose of regulating the release of scent molecules as food additives [17]. Nevertheless, the preparation process of hybrid gels is relatively complex, requiring optimization of the compatibility between different polymers and crosslinking conditions.

### 2.2. Ionic Crosslinking Mechanisms

The metal ionic crosslinking process of alginate involves the formation of “egg-box” structures, where metal ions coordinate between adjacent alginate chains [8]. The type and valency of metal ions significantly influence the properties of alginate hydrogels. Divalent cations (Ca^2+^, Ba^2+^, Sr^2+^) and trivalent cations (Fe^3+^, Al^3+^) can effectively crosslink alginate chains through coordination with carboxylate and hydroxyl groups [18]. The binding strength and crosslinking density vary depending on the ionic radius and charge density of the metal ions [19]. The resulting metal ion crosslinking network structure significantly affects the network structure, mechanical properties, and swelling behavior of the hydrogels. Higher valency metal ions typically form stronger and more stable crosslinks [20].

The exploration of the crosslinking mechanisms between different sequences on alginate chains and metal ions has been ongoing for many years. As early as the 1970s, researchers recognized the fundamental contribution of G blocks in ion binding and reported an ion affinity series as Pb^2+^ > Cu^2+^ > Cd^2+^ > Ba^2+^ > Sr^2+^ > Ca^2+^ > Co^2+^, Ni^2+^, Zn^2+^ > Mn^2+^ [21]. Mørch et al. [22] provided a reinterpretation of the affinity of various alginate sequences for certain divalent cations: GG blocks exhibit the following trend Ba^2+^ > Sr^2+^ > Ca^2+^ > > Mg^2+^, while MM blocks show Ba^2+^ > Sr^2+^ ~ Ca^2+^, and MG blocks demonstrate Ca^2+^ > Sr^2+^ ~ Ba^2+^ [22]. The concentration of metal ions significantly influences the structure and properties of alginate gel, such as density, thermal stability, diffusion resistance, and membrane-forming ability [23,24].

## 3. Alginate-Based Hydrogels Crosslinked by Different Metal Ions

The metal ion-crosslinked alginate hydrogels exhibit remarkable mechanical properties, thixotropy, and self-healing ability. The hydrogels have a porous network structure, which is influenced by the type and concentration of metal ions. There are differences in the affinity of metal ions towards alginates, with Ca^2+^, Ba^2+^, Cd^2+^, Cu^2+^, Fe^3⁺^, and Al^3⁺^ displaying higher affinities, whereas Mn^2+^, Co^2+^, Zn^2+^, and Ni^2+^ engage in weak ion crosslinking with alginates [8]. In alginate gel systems where ionic crosslinking and hydrogen bonding coexist, the mechanical properties have been investigated in relation to the strength of ionic crosslinking and hydrogen bond interactions [25]. Weak ionic crosslinking, in conjunction with hydrogen bonds, significantly enhances the tensile properties of the material. However, strong ionic crosslinking and hydrogen bonding compete with each other, leading to a swift decline in mechanical strength. By modulating the metal ions, the crosslink density and network structure of gels can be controlled to tailor their mechanical properties, structural characteristics, self-healing ability, and swelling behavior to meet diverse application requirements.

### 3.1. Alginate-Based Hydrogels Crosslinked by Ca^2+^

Calcium ions (Ca^2+^) are the most commonly used metal ion in preparing alginate-based gels. The presence of Ca^2+^ enhances the mechanical properties of alginate gels, providing them with moderate strength.

Grant et al. [26] initially proposed the binding mechanism between Ca^2+^ and alginate as the interaction of consecutive G blocks forming a buckle, two-fold structures that generate cavities for Ca^2+^ to be held in chelate binding. This configuration, known as the egg-box model, is commonly acknowledged as the predominant theory explaining the alginate gelation process.

Ramdhan et al. [27] investigated the gel strength and syneresis of alginate gels as a function of CaCl_2_ solution concentrations in an external gelation method. The results proved that the increase in the CaCl_2_ concentration resulted in a faster gelling time, increased gel strength, and a higher degree of syneresis. With the higher concentration of calcium solution, there is a proportional increase in the interaction between Ca^2+^ ions and G sequences of alginate, resulting in elevated gel strength. Li et al. Li [28] investigated the impact of Ca^2+^ on the properties of alginate-based emulsion gel. The yield stress and gel stiffness increased with higher Ca^2+^ concentrations, attributed to enhanced crosslinking within the gel network. These emulsion gels exhibited notable thixotropy and stable emulsions, showing no oil droplet coalescence post-heating or freeze–thaw cycles and having the potential application in the preparation of low-fat mayonnaise products and other similar emulsion foods.

The rheological properties of dual-network hydrogels incorporating polyacrylamide and sodium alginate under large deformations were investigated by Wang et al. [29]. The findings demonstrated that the concentration of Ca^2+^ influences the nonlinear behavior, with all gel samples displaying characteristics of strain hardening, shear thickening, and shear densification.

### 3.2. Alginate-Based Hydrogels Crosslinked by Cu^2+^

Copper ions (Cu^2+^) demonstrate superior crosslinking capabilities with alginates in comparison to Ca^2+^, resulting in the formation of a dense gel structure characterized by excellent compressive and tensile strengths. The inherent antibacterial properties of Cu^2+^ confer antimicrobial properties on the gels, showcasing potential applications in food packaging, wound dressings, and medical fields [30].

The gelation mechanism of alginate induced by Cu^2+^ differs significantly from that of calcium, as there is no preferential G or M selection for Cu^2+^ binding with alginate chains [31]. The egg-box dimer created between Cu^2+^ and alginate is consistently longer than that formed with Ca^2+^, showcasing a uniform crosslinking pattern [32]. Haug et al. [33] reported that Cu^2+^ exhibits a tenfold stronger affinity for alginate chains compared to Ca^2+^. Upon contact, Cu^2+^ swiftly binds to alginate molecules, facilitating the formation of a dense gel layer. This layer acts as a barrier, impeding the diffusion of Cu^2+^ through the gel. The increased rigidity of the gel, attributed to the dense outer layer, encloses the gel and prevents its shrinkage.

Liu et al. [34] observed that the Cu^2+^ crosslinking alginate gel exhibits small pores, a dense structure, and a high elastic modulus. Degradation experiments on the alginate gel revealed a decrease in degradation rate with an increasing Cu^2+^ concentration. Both in vitro and in vivo studies demonstrated that Cu^2+^ enhances the bone-binding properties of alginate-based gels, potentially promoting early healing of vascularization and skeletal defects, thereby supporting bone tissue engineering applications.

The presence of Cu^2+^ enhances the antibacterial properties of alginate-based gels, rendering them comparable to gels containing antibiotics [35,36]. Zhang et al. [10] proposed novel, expandable and versatile methods to prepare metal ions crosslinking alginate hydrogels with adjustable morphology, composition, and microstructure. Through the combination of sodium alginate (SA) with metal ions known for their natural antibacterial properties, an antibacterial hydrogel is formed, allowing for the gradual release of metal ions to prolong effectiveness and decrease potential toxicity. Significantly, SA has been shown to stimulate collagen deposition, thereby accelerating the wound-healing process [37]. In comparison to hydrogels crosslinked with Fe^3+^, Co^2+^, Ni^2+^, and Zn^2+^, hydrogels crosslinked with Cu^2+^ demonstrate remarkable sterilization efficacy and potent antibacterial activity against both Gram-positive and Gram-negative bacteria, including multidrug-resistant strains like Methicillin-resistant Staphylococcus aureus (MRSA) and Carbapenem-resistant Klebsiella pneumoniae (CRKP), due to their dual antibacterial mechanisms.

### 3.3. Alginate-Based Hydrogels Crosslinked by Ba^2+^

The barium ion (Ba^2+^) is a divalent cation capable of creating highly dense and strong alginates-based gels. In comparison to Ca^2+^, Ba^2+^, due to its larger ionic radius and higher affinity, it can interact with more carboxyl (COO^−^) groups [38]. This results in an enhanced crosslinking density, increased strength and stability, and reduced swelling of alginate gels.

Hassan et al. [39] demonstrated that it exhibits a significantly higher capacity and affinity within alginate compared to Ca^2+^ and Sr^2+^, facilitating easier gel formation and enhancing the stability and strength of alginate-based gels. Ba^2+^ has the ability to bind both G and M blocks but not MG blocks. The most robust Ba-alginate gels were obtained by employing 20 mM Ba^2+^ for both high-G and high-M alginate [22]. Furthermore, having a lower ion exchange equilibrium constant than Ca^2+^ and Sr^2+^, Ba^2+^ enhances the gel’s resistance to swelling, thereby preventing rapid gel degradation [39]. Santhanes et al. [40] demonstrated that Ba-alginate gels are stable in acidic and neutral pH environments but can disintegrate under alkaline conditions. The high-stability, strength, and swelling-resistant Ba-alginate has been prepared for application in dyes/salts separation [41].

### 3.4. Alginate-Based Hydrogels Crosslinked by Al^3+^

Aluminum ions (Al^3+^), as a multivalent cation, exhibit distinct effects on alginate-based gels compared to commonly used divalent cations, particularly in terms of crosslinking behavior, gel structure, and mechanical properties. Due to Al^3+^’s high charge density and strong coordination capability, it exerts a stronger influence on alginates than divalent cations.

The presence of Al^3+^ results in significantly more stable alginate gels compared to those formed with Ca^2+^ and Ba^2+^, attributed to the extended three-dimensional binding structure of Al^3+^. Despite this, the specific gelation mechanism of Al-alginate remains unclear [32]. Moreover, Al-alginate gels exhibit a higher water uptake capability than both Ca-alginate and Ba-alginate gels. In comparison to divalent ions, Al^3+^-alginate gel exhibits reduced release of trivalent ions, increased resistance to gel swelling, and diminished decline in mechanical properties [5].

The addition of Al^3+^ to hybrid gels comprising alginate, polyvinyl alcohol, aluminum-pillared montmorillonite, and calcium carbonate enhances the mechanical stability and specific surface area of alginate gel beads [42]. The alginate gel beads can be utilized to enhance Cd^2+^ adsorption in wastewater treatment.

### 3.5. Alginate-Based Hydrogels Crosslinked by Ferric Ions

Common iron ions exist in two oxidation states, divalent and trivalent. Although these two states have slightly different effects on alginate gelation, both can crosslink with alginate to create a stable gel structure.

The binding of Fe^2+^ to guluronic acid residues is not specific to the G blocks, as both G and M blocks are involved in the formation of alginate-iron hydrogels [43]. Moreover, the G blocks exhibit a higher affinity for Fe^2+^ compared to the M blocks, facilitating gelation at lower Fe^2+^ concentrations. In contrast, alginates with a higher content of M blocks require elevated levels of Fe^2+^ to establish a cohesive gel network [44]. Moreover, the crosslinking process between Fe^2+^ and alginate is notably dependent on its oxidation–reduction state. The oxidation of Fe^2+^ within Fe^2+^-alginate solution can trigger the development of Fe^3+^-alginate gel [45].

Similar to Al^3+^, Fe^3+^ can form an egg-box helical structure by interacting strongly with the carboxylate groups of alginate, leading to the creation of a stable three-dimensional interconnected gel structure [46]. Additionally, Fe^3+^ exhibits a higher affinity for binding to the deprotonated carboxyl groups of alginate compared to the protonated carboxyl groups [47]. Therefore, the Fe^3+^-alginate gel possesses an extremely robust crosslinked network, exhibiting high mechanical strength and stability [48].

### 3.6. Alginate-Based Hydrogels Crosslinked by Other Metal Ions

In addition to the aforementioned metal ions crosslinked alginate-based hydrogels, various other metal ions have been explored to prepare alginate-based gels, such as strontium ion (Sr^2+^), manganese ion (Mn^2+^), zinc ion (Zn^2+^), and silver ion (Ag^+^). Additionally, alginate hydrogels crosslinked by multiple metal ions exhibit excellent performance.

Sr^2+^ exhibits a strong binding affinity toward G blocks in the alginate chain while showing no interaction with M blocks and minimal binding with MG blocks [22]. In comparison to Ca^2+^, Sr^2+^ demonstrates increased coordination sites and enhanced binding capabilities with alginate molecules [49,50]. Consequently, Sr-alginate gels display superior chemical stability and mechanical strength when compared to Ca-alginate gels at equivalent concentrations of alginate and ions. Sr^2+^ is a non-toxic divalent cation, and the alginate gels crosslinked with strontium ions demonstrate superior mechanical properties, making them suitable for specific applications in biomedical and food sectors [51,52].

Mn^2+^ can coordinate paired G blocks akin to Ca^2+^ to create a pocket-like structure [53]. Furthermore, Mn^2+^ can interact electrostatically with M blocks, forming unstable complexes. Notably, Mn^2+^ exhibits a preference for binding to MG blocks over pure G or M blocks [54]. Nonetheless, due to its low affinity for alginate chains and tendency to form unstable hydrogels, there is a scarcity of research on Mn-alginate gels.

Zn^2+^ can bind to the carboxylate groups of G and M blocks akin to Ca^2+^. Nevertheless, the crosslinking of alginate by Zn^2+^ follows monodentate coordination, engaging solely one carboxylate oxygen atom. Consequently, Zn-alginate gels exhibit inherent looseness and weakness [55].

Due to the lack of strong polymer–ion interactions between Mg^2+^ ions and alginate, it is commonly accepted that monovalent cations and Mg^2+^ ions do not trigger strong gelation [21,56]. Zhang et al. [57] demonstrated that -OH and -COO^−^ groups are likely involved in the crosslinking of sodium alginate (SA) with Ag^+^ ions, akin to the crosslinking of SA with Ca^2+^ or other metal ions [58]. Stable Ag-alginate beads under acidic conditions were achieved when Ag^+^ concentrations were above 1.5%, showcasing promise for drug delivery purposes.

Bimetallic alginate hydrogels, created via synergistic crosslinking, exhibit enhanced attributes compared to monometallic hydrogels. These include rapid self-healing capabilities without the need for external stimuli, superior mechanical strength, antibacterial properties, increased swelling capacity, as well as magnetic and catalytic functionalities [25]. The introduction of Fe^3+^ or Cu^2+^ metal ions into alginate hydrogels, initially composed of Mn^2+^, Co^2+^, and Ni^2+^ metal ions with lower mechanical strength and higher stretchability, resulted in improved mechanical strength, decreased stretchability, and reduced water retention capability. The self-healing capability is absent in Fe-Alginate and in all bimetallic hydrogels that incorporate Fe^3+^ as one of the metal ions [59,60]. The swelling property is not observed in bimetallic hydrogels that contain Fe^3+^ or Cu^2+^. However, bimetallic hydrogels, comprising Mn^2+^, Co^2+^, and Ni^2+^, demonstrate increased swelling properties and enhanced water retention capabilities [25].

## 4. Applications

**Figure 2:**
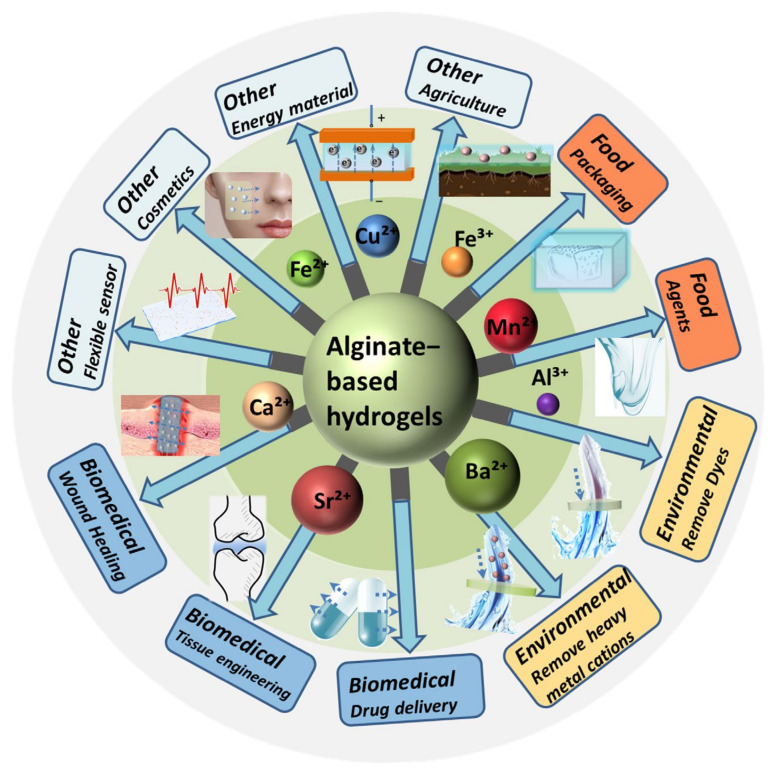
Application of alginate-based hydrogels crosslinked by metal ions.

Due to its good biodegradability, alginate hydrogels crosslinked by metal ions have shown great potential in various fields. By utilizing various metal ions as crosslinkers, alginate can generate gels with tailored properties, enabling the production of customized gels suitable for specific purposes. In the section, alginate gels employed in food, biomedical, and environmental applications are discussed (Figure 2).

### 4.1. Biomedical Applications

#### 4.1.1. Drug Delivery

Numerous drugs have been integrated into alginate matrices in different presentations for controlled release therapies, including beads, microspheres, films, and tablets [61]. Alginate gel beads are readily formed by immersing alginate droplets in a metal ion solution. This facile gelation process under mild conditions prevents the deactivation of drugs, proteins, cells, and enzymes, rendering alginate a widely utilized material for bio-encapsulation [57,62]. Alginate gels crosslinked by Ca^2+^ and Ba^2+^ have been investigated as a targeting drug delivery system in pharmaceutical productions [62,63]. The density of alginate gel is influenced by metal ion concentrations. Thus, controlled drug release can be manipulated through the adjustment of metal ion levels [64]. Furthermore, multifunctional alginate gel pharmaceutical products are being continuously developed. Kim et al. [65] reported a novel strategy to encapsulate drug and metal nanoparticles within Ca^2+^-alginate gel. This strategy, devoid of harmful reagents, is environmentally benign and offers a green approach to synthesizing diverse metal nanoparticles in a shorter time frame. The alginate gel composites could be utilized directly for creating a novel drug delivery system while facilitating in vivo imaging concurrently.

#### 4.1.2. Tissue Engineering

Alginate hydrogels serve as excellent scaffolds for tissue engineering applications due to biocompatibility, tunable mechanical properties, and cell adhesion properties [66]. Ca-alginate gel has been demonstrated to facilitate the transportation of bone-forming cells, thereby promoting bone regeneration by delivering growth factors and osteoinductive factors [67]. Investigations have shown that chondrocytes suspended in a mixture of calcium ions and alginate solution, then injected into a mold or 3D-printed create prefabricated cartilage structures for cartilage repair therapy [68]. Ca-alginate gel is applicable for crafting dental impression molds, resulting in the generation of precise tooth profiles during printing [69]. Nowadays, bio-complex alginate hydrogels are capable of mimicking the biological functions of extracellular matrix proteins, showing significant potential for on-site bone repair.

Photo-crosslinked alginate hydrogel has been extensively studied in bone tissue engineering due to its multiple benefits. Nevertheless, it exhibits drawbacks such as inadequate mechanical strength and the absence of bone induction, which limits its further application [70]. The addition of Sr^2+^ to photo-crosslinked alginate gels notably enhanced osteogenic differentiation and mineralization by promoting the expression of osteogenesis-related genes and proteins in the cells [52]. Recently, researchers developed a biomimetic Ca-alginate hydrogel to improve the osteogenic microenvironment and promote stem cell homing, indicating that the alginate hydrogels have important potential applications in bone tissue engineering and osteoporosis treatment [71]. Furthermore, the physical properties of the gels, such as swelling ratio, degradation kinetics, elastic moduli, surface morphology, and ion release, could be adjusted by altering the concentrations of Sr^2+^.

#### 4.1.3. Wound Healing

Bacterial infections in wounds remain a prominent issue in global clinical settings, posing a substantial risk to human health. Alginate gels have excellent biocompatibility, controlled degradation, and a three-dimensional porous structure, facilitating both cell growth and tissue regeneration [72,73]. The incorporation of specific metal ions can impart additional functionalities such as antibacterial properties and enhanced wound closure rates [66]. Alginate hydrogels crosslinked with metal ions like Fe^3+^, Co^2+^, Ni^2+^, Cu^2+^, and Zn^2+^ exhibit customizable morphology, composition, and microstructure [10]. The metal ions, such as Ca^2+^, Zn^2+^, and Cu^2+^, lower the environmental pH to slightly acidic values, promoting healing and increasing the antimicrobial effect against *E. coli* and *S. aureus*. Furthermore, the alginate gels conform to the wound site, prevent secondary infections, decrease inflammation, promote the proliferation of fibroblasts, increase collagen fibers, and promote the growth of new arterial and venous capillaries [74].

### 4.2. Environmental Applications

Alginate hydrogels have been explored as an effective adsorbing material for treating environmental pollution by removing various pollutants, such as heavy metals, dyes, and other sources in wastewater [75,76]. The selective adsorption of specific pollutants can be significantly enhanced by optimizing the crosslinking conditions [77,78].

The alginate gel has the capability to adsorb cationic heavy metal cations such as Ag^+^, Pb^2+^, Cd^2+^, Ni^2+^, Hg^2+^, Au^3+^, U^4+^, Cu^2+^, Ce^3+^, and chromium ions [78,79]. The adsorption behavior was predominantly dictated by both the physical and chemical attributes of the adsorbent, alongside external conditions, including pH levels and the presence of co-existing pollutants. The removal efficiency of certain heavy metal ions can even reach 100% [80].

Dyes present in wastewater could potentially pose significant risks to both the aqueous system and human health. The Ba^2+^/Ca^2+^ co-crosslinked alginate hydrogel membrane was prepared and utilized for the separation of dyes and salts in dyeing wastewater, demonstrating remarkable performance with a dye rejection rate surpassing 99.6% and a salt rejection rate under 8.2% [41]. By incorporating functional components such as metal-organic frameworks, the adsorption capacity of the alginate gels towards dyes can be enhanced [81,82]. The application of alginate gels for the remediation of arsenic contamination in groundwater has been explored [83]. Furthermore, Mn-alginate has been investigated to eliminate rebellious organic pollutants, including imidacloprid, di-2-ethylhexyl phthalate, and 4-nitrophenol [84].

### 4.3. Food Applications

In the food industry, alginate gel can be used as a preservative and packaging material. The alginate gel coating can effectively extend the shelf life of purple potato chips and reduce the growth of microorganisms [85]. In addition, alginate gel film with flaxseed oil/beeswax can significantly improve the preservation effect of mackerel [86]. Ca^2+^ crosslinked alginate-based gels, prepared by the internal gelation method, have a loose structure and large pore size [76]. Embedding plant polyphenols in the alginate-based gels can improve the function and stability of the plant polyphenols, making them suitable for fruit preservation [13].

Ca-alginate serves as a versatile thickening and stabilizing agent, offering the ability to create customized gels with varying textures and properties to meet specific needs [87]. Its exceptional gelling capabilities make it suitable for applications ranging from fluid sols to shaped, brittle, or flexible gels [88,89]. For instance, Ca-alginate can enhance the smoothness of jams or salads and stabilize ice crystal formation in ice cream [90,91].

### 4.4. Other Applications

In the agriculture field, alginate gels crosslinked with Mn^2+^, Zn^2+^, and Ca^2+^ are employed for the fabrication of biodegradable mulch films. These films exhibit superior tensile strength, elongation, water vapor transmission, and appropriate water vapor permeability [12,92]. In the energy material field, metal ion-crosslinked alginate hydrogels show promising prospects in novel energy devices such as zinc ion batteries. The SiO_2_-alginate sodium hydrogel polymer electrolyte exhibits high ionic conductivity (1.144 × 10^−2^ S·cm^−1^) and excellent mechanical strength, effectively preventing zinc dendrite growth and side reactions [93]. In the cosmetics field, alginate gels are utilized in facial masks for their ability to effectively disperse nutrients and facilitate rapid absorption. Their properties include safety, moisture retention, anti-irritation, antioxidant, anti-inflammatory, and antibacterial qualities, making them an ideal matrisx for cosmetic patches due to their excellent biodegradability [32]. The alginate hydrogel crosslinked by Fe^3+^ (1.25% *w*/*v*) demonstrates exceptional mechanical and electrical characteristics, along with self-healing abilities. This material can be utilized in the fabrication of skin-based motion sensors, proving effective in tracking the movements of human or robotic joints [94].

## 5. Conclusions

The gelation of alginate stands as a paramount functional attribute. Alginate exhibits the capability to generate gels through interactions with Ag^+^, divalent and trivalent cations, including but not limited to Pb^2+^, Cu^2+^, Cd^2+^, Ba^2+^, Sr^2+^, Ca^2+^, Co^2+^, Ni^2+^, Zn^2+^, Fe^2+^/Fe^3+^, and Al^3+^. Understanding the mechanisms of metal ion crosslinked alginate hydrogels has advanced significantly in recent years. The relationship between crosslinking mechanisms and resultant properties has been better elucidated, leading to improved control over hydrogel characteristics. Most metal ions exhibit a positive correlation with alginate-based gel properties, with the concentration of metal ions influencing the performance of the hydrogel. Metal ions effectively enhance various properties of alginate-based hydrogels, with certain ions conferring unique characteristics that expand the potential applications of the gel. However, some metal ions may impart toxicity to alginate-based hydrogels, thereby limiting their use in the field of biomedicine.

Although the alginate gels crosslinked by metal ions have been extensively researched and widely applied, numerous unresolved challenges remain. The gelation mechanisms of certain metal ions (e.g., Ag^+^, Co^2+^) with alginate are still under debate. Furthermore, the relationship between crosslinking mechanisms, gel structure, and material properties lacks comprehensive understanding, hindering precise tuning and optimization of gel performance. Traditional alginate gels suffer from low mechanical strength, which restricts their application in scenarios requiring high mechanical robustness. Therefore, further exploration is needed to elucidate the co-gelation mechanisms of alginate with other polymers, aiming to fabricate high-strength hydrogels.

In future research, it is essential to focus on investigating the crosslinking mechanism and developing functional alginate hydrogels. Research on crosslinking mechanisms aids in achieving precise control over the crosslinking density, advancing dual and triple metal ion crosslinking systems for the fabrication of alginate gels with customizable properties. By investigating the incorporation of other components in the metal ions crosslinking alginate gel, the biodegradable functional alginate gels can be applied more widely, such as environmental and smart responsive materials for biomedical and packaging, gels with complex spatial structures for industrial engineering, and superabsorbent gels for solar water purification. Metal ions exert a significant influence on alginate-based hydrogels, holding great potential for enhancing various properties of the gels and expanding their range of applications. These efforts aim to facilitate the production of a broader array of biodegradable alginate applications, thereby broadening their scope of practical use.

## Data Availability

No new data were created or analyzed in this study.
